# Effects of Customized Long-Message Service and Phone-Based Health-Coaching on Elderly People with Hypertension

**Published:** 2019-04

**Authors:** Myoungsuk KIM

**Affiliations:** College of Nursing, Kangwon National University, Chuncheon-si, Gangwon-do, Republic of Korea

**Keywords:** Hypertension, Medication adherence, Self-efficacy, Self-management

## Abstract

**Background:**

We aimed to develop long-message services (LMS) and phone-based health-coaching for community-dwelling seniors diagnosed with hypertension and assess the effects of the programs implemented both separately and together. These programs are easily applicable to seniors diagnosed with hypertension and will help control their blood pressure (BP) in a practical manner.

**Methods:**

We conducted a single-blinded, randomized, controlled pragmatic trial. Individuals aged 65 years or older with hypertension at two senior welfare centers in Seoul, South Korea, who were able to take phone calls and check text messages were enrolled. The study included 124 participants: 31 in the control group, 30 in the health-coaching group, 32 in the LMS group, and 31 in the health-coaching-with-LMS group.

**Results:**

Phone-based health-coaching with LMS was effective in improving medication adherence, hypertension self-efficacy, and self-management behavior and decreasing systolic BP as compared to LMS only. There were also improvements in medication adherence, hypertension-related knowledge, hypertension self-efficacy, self-management behavior, and systolic BP in the LMS group as compared to the control group.

**Conclusion:**

Using phone-based health-coaching with LMS was effective for managing hypertension in community-dwelling seniors diagnosed with hypertension and could become a useful intervention method.

## Introduction

Hypertension is the ninth most common cause of death among adults in South Korea; about 11 per 100,000 people will die from a hypertension-related disease (1). The overall prevalence of hypertension is 35.0% in men and 22.9% in women; this increases with age to 55.9% in men and 46.2% in women aged between 60–69 years and 64.2% in men and 72.5% in women aged 70 years or older (2). Furthermore, hypertension is the most important and powerful risk factor for cardiovascular diseases, and the risk increases with the overlapping presence of several factors (3). For example, hypertension with uncontrollable blood pressure (BP) causes 20% of premature cardiovascular-related deaths (4).

However, if patients with hypertension control their BP, they can reduce the complications of hypertension (5), such as cardiovascular diseases or stroke. The Eighth Report of the Joint National Committee (JNC8) guideline emphasizes the importance of self-management behaviors including diet, weight control, and regular exercise to control BP and reduce use of antihypertensive medication (6).

There are various BP control programs that improve self-management behavior in patients with hypertension. Information communication technology, such as text messages, could be utilized to prevent and manage chronic diseases, such as hypertension, owing to its ease-of-use and cost effectiveness (7–8). Health-coaching is becoming an increasingly popular means of improving chronic diseases (9). However, the only studies conducted in Korea include one in which participants measured their BP and sent these measurements via text messages (7), and one wherein only five text messages that included health information were used over a 12-week period (10); this shows that there is a severe lack of research examining the use of text messaging among patients with hypertension. Furthermore, only one study has examined the effects of phone-based health-coaching on patients with hypertension in Korea (11). Although studies in other countries have examined the use of text messages for patients with hypertension (12–14) or implemented health-coaching (9, 15), research on phone-based health-coaching is scarce. In addition, few studies have examined the effects of implementation of text messaging or phone-based health-coaching on seniors. While this population could experience difficulty in using cellphones because they are unfamiliar with these platforms, cellphone-based health management could be useful to elderly patients for managing chronic diseases (16). Therefore, assessing the effects of text messaging and/or phone-based health-coaching on seniors could help them practically manage hypertension. As individual patient-tailored content is more effective than simple text message reminders (8), it is important to implement customized LMS based on participants’ diseases and lifestyles and verify their effects.

This study developed customized LMS and phone-based health-coaching and implemented them both separately and together in a randomized controlled trial involving community-dwelling seniors diagnosed with hypertension, and examined the effects of the coaching on hypertension self-efficacy, hypertension self-management, medication adherence, hypertension-related knowledge, and BP. The study aimed to develop practically helpful and applicable intervention and assess the effects of these programs on community-dwelling seniors diagnosed with hypertension.

## Methods

### Study Design

The study customized LMS and/or phone-based health-coaching provided to community-dwelling seniors with hypertension.

Data were collected, and the intervention began after receiving approval from the university’s institutional review board (IRB No. SSWUIRB 2017–068). Participants were recruited through an announcement on bulletin boards at senior welfare centers and they participated in the study voluntarily. The study purpose and method were explained both verbally and in writing, and all participants provided written informed consent.

### Settings and Participants

“The study was conducted at two senior welfare centers, in northern or southern Seoul, South Korea, from July 30 to September 29, 2018.

Participants who were seniors aged 65 years or older, able to receive phone calls and text messages, prescribed antihypertensive medication (to verify a hypertension diagnosis), able to communicate, and without visual impairment were included in the study. However, individuals with mental illnesses; neurological diseases, and a receipt of treatment for severe depression or anxiety were excluded from the study.

Of the 149 eligible participants from two senior welfare centers, after excluding three participants who did not satisfy the inclusion criteria and two who refused consent, data for 144 are enrolled. After completing enrollment and baseline measurements, participants were randomly assigned 36 people to each group. In the health-coaching-with-LMS condition, two participants withdrew over a phone call, and phone numbers were incorrect for two participants. In the health-coaching condition, one participant refused to participate, and phone numbers were incorrect for two. In the LMS condition, two participants refused to participate. During intervention, one participant from the health-coaching-with-LMS group was hospitalized; moreover, three participants from the health-coaching group, two from the LMS group, and five from the control group were excluded because their BP could not be measured. Therefore, the final groups included 31 participants in the control group, 30 in the health-coaching group, 32 in the LMS group, and 31 in the health-coaching-with-LMS group.

### Enrollment, Randomization, and Blinding

An individual who was not affiliated with our study used a random allocation program (https://www.randomizer.org/) to assign participants. They completed a survey and underwent BP measurement; participants were then allocated to a group. The research assistants who measured the participants’ BP and conducted the surveys before and after the intervention were blind to the study arms to which participants were assigned. The nurses who performed health-coaching were also unaware of whether the participants were part of the health-coaching-with-LMS or health-coaching arm. However, because the nature of the study required participants to be aware of the intervention in which they would participate, they could not be blinded to this information.

### Experimental Procedure

Health-coaching was divided into health information and coaching methods. Health information was developed based on the JNC8 and the hypertension treatment guidelines published by the Korean Society of Hypertension. Health information included the definitions of hypertension, complications, diet, medication, exercise, weight, stress, drinking, smoking, and sleep. The validity of this content was examined by a group of experts (2 doctor, and 3 nursing professors); the content validity index was high for all items, at 0.89–1.00, indicating that detailed opinions had been obtained. The coaching method was developed based on Cox’s Interaction Model of Client Health Behavior (IMCHB) (17). The details are shown in Table 1.

**Table 1:** Phone-based health-coaching process

***Session***	***Content outline***	***IMCHB sub-domain***
1^st^ week	Pretest (general characteristics) Program introduction	Intrinsic motivation
	Definition of hypertension and complications Planning and deciding on a method for managing complications Sharing the difficulties	Health information Decisional control Affective support
2^nd^ week	Healthy diet Planning and deciding on a method for managing diet Sharing the difficulties	Health information Decisional control Affective support
3^rd^ week	Taking medicine Planning and deciding on a method for managing medication Sharing the difficulties	Health information Decisional control Affective support
4^th^ week	Regular exercise Planning and deciding on an exercise method Sharing the difficulties	Health information Decisional control Affective support
5^th^ week	Weight control Planning and deciding on a weight control method Sharing the difficulties	Health information Decisional control Affective support
6^th^ week	Stress management Planning and deciding on a method for stress management Sharing the difficulties	Health information Decisional control Affective support
7^th^ week	Non-smoking and non-drinking Planning and deciding on a non-smoking and non-drinking Sharing the difficulties	Health information Decisional control Affective support
8^th^ week	Healthy sleep Planning and deciding on a health sleep method Sharing the difficulties Post-test	Health information Decisional control Affective support Health outcomes

IMCHB: Cox’s Interaction Model of Client Health Behavior

The LMS was also developed based on the JNC8 and the hypertension treatment guidelines. The LMS content included information such as provided by health-coaching. Customized content was developed for 16 algorithms based on whether the participants had comorbidities, such as diabetes, hyperlipidemia, and obesity, and lifestyle habits, such as smoking or drinking. Content was designed to be sent 1–2 times per day, and 3 times per week for 8 weeks. The length of the LMS was 163–230 bytes. The LMS was revised by 2 doctors of family or internal medicine, and 2 nurses.

Phone-based health-coaching was provided by a nurse who had completed over 40 hours of health management training from a healthcare service company. Health-coaching was provided for 30 minutes once a week. For the LMS, an information-processing professional used an automatic delivery system to send each participant’s individual algorithm automatically on a designated date.

### Measures

The dependent variables were assessed at the enrollment stage and after the intervention.

Hypertension self-efficacy was measured using a tool measured on a 10–100-point scale developed by Park and Hong (18). Based on average scores for 10 items, a higher self-efficacy score indicated greater confidence regarding self-management behavior. Cronbach’s α was 0.72 when the tool was developed; in this study, it was 0.82.

Hypertension self-management behavior was measured using a 16-item tool developed by Lee (19). Responses were measured using a 5-point scale; higher scores indicated higher levels of self-management. Cronbach’s α was 0.72 when the tool was developed; in this study, it was 0.78.

Medication adherence was measured using scale developed by Morisky et al. (20) and was adapted for use in Korean populations by Han (21). Higher scores for the four items indicate stronger medication adherence. Cronbach’s α was 0.61 when the tool was developed; in this study, it was 0.76.

Hypertension-related knowledge was measured using a 12-item tool developed by Park and Hong (18), which was revised by Oh (22). Responses were measured using a 2-point scale. Higher scores indicate greater knowledge regarding hypertension. In the study by Oh (22), Kuder Richardson reliability was 0.87; Cronbach’s α in this study was 0.89.

BP was measured using an electronic BP gauge (UA-767PBT, A&D Medical, Saitama, Japan) while participants were relaxed, and patients who needed to use the bathroom were allowed to do so beforehand. Participants’ arms were placed at chest level for measurement.

### Statistical analysis

Data were analyzed using IBM SPSS Statistics ver. 21.0 (IBM Co., Armonk, NY, USA). To test for prehomogeneity in the general characteristics of each group, χ^2^ and Fisher’s exact tests were performed. A one-way analysis of variance was performed for prehomogeneity testing on dependent variables and verification of the effects of changes between baseline and 8-week follow-up measurement. Statistical significance was set at *P* < 0.05.

## Results

Table 2 shows the participants’ general characteristics and the results of the homogeneity test between the experimental and control groups. The results showed no significant differences in age, sex, education, duration of hypertension diagnosis, exercise frequency, smoking habits, drinking habits, or BMI between the groups, confirming participant homogeneity (Table 2). There were no significant differences in dependent variables between groups before intervention, ensuring between-group homogeneity (*p* > 0.05).

**Table 2:** Homogeneity of participants’ general characteristics and dependent variables before intervention

***Characteristics***		***Control (N=31)***	***Health-coaching (N =30)***	***LMS (N =32)***	***Health-coaching-with-LMS (N =31)***	***χ^2^ or F***	***P***
		***N (%) or Mean ± standard deviation***					
Age (yr)		77.70 ± 6.92	77.50 ± 6.38	77.21 ± 6.69	78.25 ± 6.61	0.12	0.943
Gender	Male	20 (64.5)	19 (63.3)	12 (37.5)	17 (54.8)	5.93	0.116
	Female	11(35.5)	11 (36.7)	20 (62.5)	14 (45.2)		
Education level	Illiteracy	1 (3.2)	3 (10.0)	1 (3.1)	3 (9.7)	10.18	0.602
	Elementary school	3 (9.7)	4 (13.3)	9 (28.1)	4 (12.9)		
	Middle school	7 (22.6)	5 (16.7)	4 (12.5)	9 (29.0)		
	High school	13 (41.9)	11 (36.7)	13 (40.6)	12 (38.7)		
	≥ College graduate	7 (22.6)	7 (23.3)	5 (15.6)	3 (9.7)		
Duration of hypertension diagnosis (year)		14.38 ± 10.01	13.73 ± 8.40	13.87 ± 10.58	11.61 ± 8.07	0.49	0.686
Exercise	No	4 (12.9)	5 (16.7)	5 (15.6)	3 (9.7)	7.63	0.578
	1∼2 times/week	6 (19.4)	1 (3.3)	5 (15.6)	7 (22.6)		
	3∼4 times/week	8 (25.8)	10 (33.3)	12 (37.5)	11 (35.5)		
	≥ 5 times/week	13 (41.9)	14 (46.7)	10 (31.3)	10 (32.3)		
Smoking	Yes	3 (9.7)	1 (3.3)	3 (9.4)	1 (3.2)	1.94†	0.630
	No	28 (90.3)	29 (96.7)	29 (90.6)	30 (96.8)		
Alcohol use	Yes	7 (22.6)	8 (26.7)	4 (12.5)	10 (32.2)	3.67	0.313
	No	24 (77.4)	22 (73.3)	28 (87.5)	21 (67.7)		
BMI		21.30 ± 2.19	21.36 ± 2.31	22.44 ± 2.08	21.67 ± 2.00	1.85	0.142
Hypertension self-efficacy		7.66 ± 1.23	7.85 ± 0.91	7.61 ± 1.08	7.40 ± 1.06	0.86	0.460
Self-management behavior		60.32 ± 10.01	62.20 ± 8.35	64.40 ± 7.53	59.19 ± 9.18	2.11	0.102
Hypertension-related knowledge		7.93 ± 1.91	7.66 ± 1.91	7.90 ± 1.65	7.87 ± 1.91	0.15	0.927
Medication adherence		0.74 ± 1.06	0.66 ± 0.84	0.59 ± 1.01	0.74 ± 1.15	0.49	0.921
Systolic BP		140.03 ± 11.60	141.16 ± 18.28	142.90 ± 12.08	142.00 ± 13.33	0.23	0.868
Diastolic BP		88.06 ± 6.44	87.10 ± 10.54	87.25 ± 7.87	86.61 ± 5.63	0.51	0.676

LMS: long-message services

† Tested using the Fisher’s exact test

Table 3 shows the results of verifying differences between dependent variables after intervention. There were significant differences in hypertension self-efficacy, hypertension self-management, medication adherence, hypertension-related knowledge, and BP between the four groups.

**Table 3:** Comparison of dependent variables after intervention

***Variables***	***Control (N=31)^a^***	***Health-coaching (N=30)^b^***	***LMS (N=32)^c^***	***Health-coaching-with-LMS (N=31)^d^***	***F***	***P***	***Scheffe post-hoc***
	***Mean ± standard deviation***						
Hypertension self-efficacy	7.77 ± 0.90	8.34 ± 0.81	7.52 ± 0.78	8.68 ± 0.69	13.63	<0.001***	b, d>c>a
Self-management behavior	61.93 ± 7.01	71.80 ± 3.91	67.21 ± 5.77	73.00 ± 4.25	26.77	<0.001***	b, d >c>a
Hypertension-related knowledge	7.77 ± 1.54	8.63 ± 0.92	8.59 ± 0.97	9.45 ± 0.88	11.65	<0.001***	d>b, c>a
Medication adherence	0.87 ± 1.08	2.503 ± 1.22	1.87 ± 1.38	2.87 ± 1.11	16.21	<0.001***	b, d >c>a
Systolic BP	143.58 ± 9.49	132.53 ± 8.92	139.15 ± 7.51	133.61 ± 7.32	10.11	<0.001***	b, d >c>a
Diastolic BP	87.88 ± 8.12	83.60 ± 9.05	85.75 ± 6.72	82.29 ± 4.79	3.42	0.019*	d>a

LMS: long-message services //

* *P*<0.05,

*** *P*<0.001, as tested using the one-way analysis of variance

Through Scheffe post-hoc, there were significant differences in hypertension self-efficacy, hypertension self-management, medication adherence, and systolic BP between the health-coaching-with-LMS and health-coaching groups as compared to the LMS and control groups (*P*<0.05). There was a significant increase in hypertension-related knowledge in the health-coaching-with-LMS group as compared to the other groups. However, there were significant differences in hypertension self-efficacy, hypertension self-management behavior, medication adherence, hypertension-related knowledge, and systolic BP between the LMS-only and control groups. There was a significant decrease in diastolic BP only the health-coaching-with-LMS and control groups.

## Discussion

This study developed a customized phone-based health-coaching and LMS method for community-dwelling seniors diagnosed with hypertension to assess the effects of the programs on hypertension self-efficacy, hypertension self-management, medication adherence, hypertension-related knowledge, and BP, when implemented both separately and together. The effects were more significant for the health-coaching-with-LMS group than for the LMS and control groups. This was the first randomized controlled trial implementing phone-based health-coaching and LMS, both separately and together, community-dwelling seniors who were diagnosed with hypertension, and examining the effects of these interventions. In addition, this study also assessed the effectiveness of customized LMS rather than simple text message reminders or phone-based health-coaching for seniors who have difficulty using mobile phones, and examined the practical effects of these methods on hypertension management for seniors.

The health-coaching-with-LMS and health-coaching methods were more effective than using LMS-only, with respect to hypertension self-efficacy, self-management behavior, medication adherence, and systolic BP; there was no major difference in effects between health-coaching and health-coaching-with-LMS. This shows that phone-based health-coaching is more effective than LMS for hypertension management among seniors and suggests that phone-based health-coaching must be integrated and implemented, more than LMS, among seniors in the future for hypertension management.

Although LMS is not as effective as phone-based health-coaching, it was more effective than control group with respect to hypertension self-efficacy, self-management behavior, hypertension-related knowledge, medication adherence, and systolic BP. This is consistent with the results of previous studies that revealed an increase in medication adherence and a decrease in BP among seniors and adults after they started receiving short message service (SMS) reminders on antihypertensive medication (13, 23). In other words, this study showed that LMS like SMS, is effective for hypertension management. However, a comparative study is necessary in the future to examine whether simple text message or LMS reminders with detailed customized health information is more effective. In addition, the duration of sending SMS reminders to change participants’ lifestyle habits varied across previous studies, ranging from 6 weeks to 12 months (24), and the frequency also differed from twice a week (25) to daily (13). Although this study, which was conducted over 8 weeks, was effective in changing the health behavior of seniors, follow-up is required to examine how effective these methods are in the long-term. Research must also be conducted to determine the differences in effectiveness based on the weekly frequency of LMS.

Conversely, studies report that there was no significant difference in medication compliance after sending SMS to hypertension patients 2 days/week for 4 months compared to control (12). In most studies involving SMS, the intervention period was 3–12 months (12, 13, 23); this study was comparatively shorter (2 months). However, the LMS groups exhibited improvements in medication adherence, systolic BP, hypertension self-efficacy, self-management behavior, and hypertension-related knowledge as compared to the control group. Although follow-up is required to identify the element that led to these results, the provision of customized health information through LMS is believed to have impacted the effects. In previous studies, health messages tailored for participants through text messages were more effective at changing behaviors than untailored messages, and participants were more willing to participate (26–28).

The content of these tailored SMS reminders included customized medication adherence, such as the drug name, dosage, strength, and frequency for each participant (13). However, the LMS in this study also provided customized health information based on participants’ comorbidities and lifestyle habits, in addition to the customized content from previous studies; this is believed to have impacted the participants’ health outcomes positively.

Regarding phone-based health-coaching, medication adherence improved when medical assistants provided phone-based health-coaching once every 3 months or once a month over 12 months for patients aged between 18–75 years, with chronic diseases such as diabetes, hypertension, and hyperlipidemia (9). However, this study provided more frequent, focused, and systematic coaching than previous studies by coaching the participants once a week for a short period of 8 weeks, proving that short-term phone-based health-coaching can also be effective. However, additional research is required to understand the differences related to intervention duration and frequency. The uniqueness of this study is that it provided customized coaching. In previous studies, 6 monthly education sessions were provided to hypertension patients by a nurse during home and clinic visits; education on healthy lifestyle behaviors was more effective at reducing BP than medication-adherence education alone (29). This suggests that, rather than simple reminders, customized coaching based on the participants’ comorbidities and lifestyle habits had a significant impact on their health outcomes.

In another study of patients with hypertension, participants were coached on action plans, disease-specific knowledge, and medication adherence by a health coach at a clinic once every 3 months, over a period of 12 months; however, there was no reduction in BP in the experimental group receiving health-coaching compared to the control group (30). Hence, more research that inspect various aspects, such as whether the difference stems from visit-based or phone-based health-coaching, differing intervention periods, or differences in customized health-coaching, are needed. Our results showed that phone-based health-coaching, which is easier than visit-based coaching, is also effective for managing BP among seniors in the community.

This study had several limitations. First, because the effects were measured over a short 8-week period, the long-term effects could not be verified. Second, although research assistants were blind during arm assignment of participants, health-coaching intervention, and survey, because participants were aware of which intervention they were receiving and could not be blind, the study could not be double-blinded. Third, the participants were seniors diagnosed with hypertension at two senior welfare centers in Seoul, South Korea, which makes it difficult to generalize our findings to all participants.

## Conclusion

This study developed a phone-based health-coaching and LMS method for community-dwelling seniors diagnosed with hypertension to assess the effects of intervention on hypertension self-efficacy, self-management, hypertension-related knowledge, and BP, using either or both methods, and found that the effects were more significant for the health-coaching-with-LMS and health-coaching groups for medication adherence, hypertension self-efficacy, self-management behavior, and systolic BP, than for the LMS and control groups. Phone-based health-coaching was more effective for hypertension management among community-dwelling seniors diagnosed with hypertension. Therefore, it was concluded that this method could be useful for intervention.

## Ethical Considerations

Ethical issues (Including plagiarism, informed consent, misconduct, data fabrication and/or falsification, double publication and/or submission, redundancy, etc.) have been completely observed by the authors.
